# *Shigella* Antimicrobial Drug Resistance Mechanisms, 2004–2014

**DOI:** 10.3201/eid2206.152088

**Published:** 2016-06

**Authors:** Magdalena Nüesch-Inderbinen, Nicole Heini, Katrin Zurfluh, Denise Althaus, Herbert Hächler, Roger Stephan

**Affiliations:** University of Zurich, Zurich, Switzerland

**Keywords:** Shigella, antimicrobial treatment, multidrug resistance, resistance genes, ciprofloxacin, cephalosporins, azithromycin, antimicrobial resistance, bacteria

## Abstract

To determine antimicrobial drug resistance mechanisms of *Shigella *spp., we analyzed 344 isolates collected in Switzerland during 2004–2014. Overall, 78.5% of isolates were multidrug resistant; 10.5% were ciprofloxacin resistant; and 2% harbored *mph(A)*, a plasmid-mediated gene that confers reduced susceptibility to azithromycin, a last-resort antimicrobial agent for shigellosis.

*Shigella* spp. are the etiologic agents of acute invasive intestinal infections clinically manifested by watery or bloody diarrhea. Shigellosis represents a major burden of disease, especially in developing countries, and is estimated to affect at least 80 million persons, predominantly children, each year (*1*). Disease may be caused by any of the 4 *Shigella* species: *S. dysenteriae, S. flexneri*, *S. boydii*,* and S. sonnei*. In industrialized countries, the most common species is* S. sonnei,* but this species is spreading intercontinentally to developing countries as a single, rapidly evolving lineage (*2*). By contrast, in developing countries, the predominant species is *S. flexneri*, which is characterized by long-term persistence of sublineages in shigellosis-endemic regions with inadequate hygienic conditions and unsafe water supplies (*3*). More rarely isolated are *S. dysenteriae*, responsible for large epidemics in the past, and *S. boydii* (*4*). Although shigellosis is principally a self-limiting disease, the World Health Organization guidelines recommend antimicrobial drug treatment as a means of reducing deaths, disease symptoms, and organism-excretion time; the current drug of choice is ciprofloxacin (*1*). Of growing concern is multidrug resistance, and in particular the increasing rate of resistance to ciprofloxacin reported for *Shigella* isolates from Asian and African regions (*5*). Furthermore, resistance to recommended second-line antimicrobial drugs, such as the third-generation cephalosporin ceftriaxone and the macrolide azithromycin, is emerging (*1*).

## The Study

To determine antimicrobial drug resistance profiles, we analyzed clinical isolates representing 344 *Shigella* spp. collected during 2004–2014. We focused on molecular resistance mechanisms that promote resistance to currently recommended antimicrobial drugs.

We performed susceptibility testing by using the Kirby–Bauer disk-diffusion method. Results were interpreted according to Clinical and Laboratory Standards Institute performance standards (*6*). All 344 isolates were screened for plasmid-mediated quinolone resistance (PMQR) genes (*7*). A subset of 34 isolates eliciting reduced susceptibility to nalidixic acid, ciprofloxacin, or both, and representing different years of isolation was subjected to PCR-based detection of mutations in the quinolone resistance-determining regions (QRDRs) of the *gyrA* and *parC* genes (*7*). Isolates showing an extended-spectrum β-lactamase (ESBL) phenotype were screened by PCR for the presence of genes belonging to the *bla*_TEM_, *bla*_SHV_, and *bla*_CTX-M_ families, by using primers described previously (*8*). All 344 isolates were analyzed for *mph(A)* by PCR by using previously published primers (*9*). Resulting amplicons were purified and sequenced. For database searches, we used blastn (http://www.ncbi.nlm.nih.gov/blast/).

Multidrug resistance was defined as resistance to >3 classes of antimicrobial agents. Multidrug resistance was detected in 150 (83.8%) of the *S. sonnei*, 84 (78.5%) of the *S. flexneri*, 20 (60.6%) of the *S. dysenteriae*, and 16 (64%) of the *S. boydii* isolates (Table 1).

**Table 1:** Antimicrobial drug resistance of 344 *Shigella* spp. isolates, Switzerland, 2004–2014

Agent	No. (%) isolates			
	*S. sonnei*, n = 179	*S. flexneri*, n = 107	*S. dysenteriae*, n = 33	*S. boydii*, n = 25
Ampicllin	31 17.3)	73 (68.2)	19 (57.6)	12 (48)
Amoxicillin/clavulanic acid	2 (1.1)	1 (0.9)	0	0 (0)
Cephalothin	12 (6.7)	0	0	0
Cefotaxime	8 (4.5)	0	0	0
Nalidixic acid	49 (27.4)	15 (14)	2 (6)	2 (8)
Ciprofloxacin	27 (15)	9 (8.4)	0	0
Azithromycin*	2 (1.1)	5 (4.7)	0	0
Trimethoprim	172 (96)	70 (65.4)	20 (60.6)	15 (60)
Sulfamethoxazole	151 (84.4)	71 (66.4)	19 (57.6)	16 (64)
Kanamycin	1 (0.5)	1 (0.9)	0	0
Gentamicin	4 (2.2)	0	0	0
Streptomycin	163 (91)	81 (75.7)	24 (72.7)	18 (72)
Tetracycline	145 (81)	83 (77.6)	22 (66.6)	13 (52)
Chloramphenicol	6 (3.4)	56 (52.3)	9 (27.3)	2 (8)

*For azithromycin, no Clinical and Laboratory Standards Institute breakpoints for *Enterobacteriaceae* exist. Isolates harboring *mph(A)* were regarded as resistant.

Resistance to nalidixic acid was detected in all species, but none of the *S. dysenteriae* and *S. boydii* isolates were resistant to ciprofloxacin (Table 1). The time distribution and the frequency of ciprofloxacin-resistant *S. sonnei* isolates showed a rising tendency (Figure). A similar tendency was noted for ciprofloxacin-resistant *S. flexneri* isolates, which, however, revealed higher variability throughout the study period (Figure). No ciprofloxacin-resistant isolates were found before 2008. In total, 27 (15%) *S. sonnei* and 9 (8.4%) *S. flexneri* isolates were resistant to ciprofloxacin.

**Figure:**
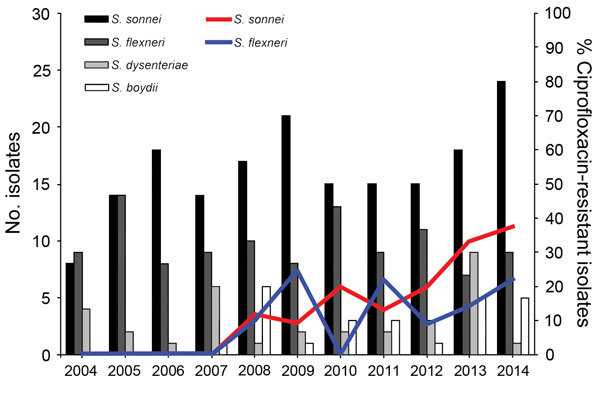
*Shigella* spp. isolated in Switzerland, 2004–2014, and percentages of ciprofloxacin-resistant *S. sonnei* and *S. flexneri*.

The *qnrS1* gene was found in 13 (3.8%) of the strains: 4 *S. dysenteriae*, 4 *S. flexneri*, 4 *S. boydii*, and 1 *S. sonnei*. Other PMQR genes included *qnrB19*, detected in *S. sonnei* (n = 1), and *qnrB4*, detected in combination with *qepA* in *S. sonnei* (n = 1). Of the 15 PMQR-positive isolates, only 2 were resistant to nalidixic acid and ciprofloxacin, illustrating the potential for development of resistance in susceptible strains (Table 2).

**Table 2:** Presence of PMQR determinants, amino acid changes in QRDR, ESBLs and the macrolide resistance gene *mph(A)* in *Shigella* spp. isolated in Switzerland, 2004–2014, and MICs of azithromycin against isolates containing mph(A)*

Year of isolation, strain	*Shigella *species	PMQR	QRDR					ESBL	*mph(A)*	MIC, μg/mL
			GyrA			ParC				
			Ser83	Asp87		Ser80	Ala85			
2004										
412–04	* sonnei*	–	Leu	wt		wt	wt	ND	–	ND
1497–04	* flexneri*	–	ND	ND		ND	ND	ND	+	24
2005										
1826–05	* sonnei*	–	Leu	wt		wt	wt	ND	–	ND
263–05	* flexneri*	–	Leu	wt		wt	wt	ND	–	ND
693–05	* flexneri*	–	ND	ND		ND	ND	ND	+	32
1319–05	* sonnei*	–	ND	ND		ND	ND	ND	+	256
1742–05	* flexneri*	–	ND	ND		ND	ND	ND	+	12
2006										
1920–06	* flexneri*	–	Leu	wt		wt	wt	ND	–	ND
1549–06	* sonnei*	–	Leu	wt		wt	wt	ND	–	ND
2007										
2389–07	* sonnei*	–	Leu	wt		wt	wt	ND	–	ND
1374–07	* flexneri*	–	Leu	wt		wt	wt	ND	–	ND
2008										
2372–08	* boydii*	–	Leu	wt		wt	wt	ND	–	ND
2157–08	* sonnei*	–	Leu	Gly		Ile	wt	ND	–	ND
2134–08	* boydii*	–	Leu	wt		wt	Ser	ND	–	ND
822–08	* flexneri*	–	Leu	Gly		Ile	wt	ND	–	ND
959–08	* boydii*	*qnrS1*	ND	ND		ND	ND	ND	–	ND
2009										
09–2751	* flexneri*	–	Leu	wt		wt	wt	ND	–	ND
09–2192	* sonnei*	–	Leu	wt		wt	wt	CTX-M-14	–	ND
09–1001	* dysenteriae*	–	wt	Asn		wt	wt	ND	–	ND
09–684	* sonnei*	–	Leu	Gly		Ile	wt	ND	–	ND
006–09	* flexneri*	–	Leu	Asn		Ile	wt	ND	–	ND
375–09	* flexneri*	*qnrS1*	ND	ND		ND	ND	ND	–	ND
09–736	* sonnei*	–	ND	ND		ND	ND	CTX-M-15	–	ND
2010										
10–1982	* sonnei*	–	wt	Tyr		wt	wt	ND	–	ND
10–1935	* boydii*	–	Leu	wt		wt	wt	ND	–	ND
10–1557	* dysenteriae*	–	Leu	wt		wt	wt	ND	–	ND
10–433	* sonnei*	–	Leu	Gly		Ile	wt	ND	–	ND
10–383	* flexneri*	–	Leu	wt		wt	wt	ND	–	ND
10–1166	* flexneri*	*qnrS1*	ND	ND		ND	ND	ND	–	ND
10–929	* boydii*	*qnrS1*	ND	ND		ND	ND	ND	–	ND
10–338	* dysenteriae*	*qnrS1*	ND	ND		ND	ND	ND	–	ND
2011										
11–0683	* sonnei*	–	wt	Tyr		wt	wt	CTX-M-15	–	ND
11–0616	* flexneri*	–	wt	Tyr		wt	wt	ND	–	ND
11–0162	* sonnei*	–	Leu	Gly		Ile	wt	ND	–	ND
11–0029	* flexneri*	–	Leu	Gly		Ile	wt	ND	–	ND
11–1023	* sonnei*	–	ND	ND		ND	ND	CTX-M-15	–	ND
2012										
12–0580	* sonnei*	–	Leu	Gly		Ile	wt	ND	–	ND
12–0273	* flexneri*	–	Leu	Gly		Ile	wt	ND	–	ND
12–0094	* dysenteriae*	–	Leu	wt		wt	wt	ND	–	ND
12–0573	* dysenteriae*	*qnrS1*	ND	ND		ND	ND	ND	–	ND
12–0087	* flexneri*	*qnrS1*	ND	ND		ND	ND	ND	–	ND
2013										
13–2304	* flexneri*	–	Leu	Gly		Ile	wt	ND	–	ND
13–1996	* boydii*	–	Leu	wt		wt	Ser	ND	–	ND
13–1909	* sonnei*	–	wt	Tyr		wt	wt	CTX-M-14	–	ND
13–0136	* flexneri*	*qnrS1*	ND	ND		ND	ND	ND	–	ND
13–1494	* dysenteriae*	*qnrS1*	ND	ND		ND	ND	ND	–	ND
13–1295	* sonnei*	*qnrS1*	ND	ND		ND	ND	ND	–	ND
13–1205	* boydii*	*qnrS1*	ND	ND		ND	ND	ND	–	ND
13–2356	* boydii*	*qnrS1*	ND	ND		ND	ND	ND	–	ND
2014										
14–2394	* sonnei*	–	Leu	Gly		Ile	wt	ND	–	ND
14–0754	* flexneri*	–	Leu	Asn		Ile	wt	ND	+	16
14–0369	* sonnei*	–	Leu	Gly		Ile	wt	ND	–	ND
14–1127	* sonnei*	–	ND	ND		ND	ND	CTX-M-15	–	ND
14–1843	* flexneri*	–	ND	ND		ND	ND	ND	+	48
14–1990	* sonnei*	*qnrB4*, *qepA*	ND	ND		ND	ND	ND	+	>256
14–1929	* sonnei*	*qnrB19*	ND	ND		ND	ND	ND	–	ND
14–1570	* dysenteriae*	*qnrS1*	ND	ND		ND	ND	ND	–	ND
14–1495	* sonnei*	–	ND	ND		ND	ND	CTX-M-3	–	ND
14–1820	* sonnei*	*–*	ND	ND		ND	ND	CTX-M-15	–	ND

*ESBL, extended-spectrum β-lactamase; ND, not determined; PMQR, plasmid-mediated quinolone resistance; QRDR, quinolone resistance determining regions; wt, wild type; –, negative; +, positive.

Most of the 34 isolates analyzed for mutations in their QRDR carried mutations in the *gyrA* and *parC* genes (Table 2). Most frequently observed was the first-step amino acid substitution within GyrA at Ser83Leu (n = 14), which was associated with resistance to nalidixic acid. The double substitutions within GyrA at Ser83Leu/Asp87Gly (n = 11) and Ser83Leu/Asp87Asn (n = 2) occurred invariably in combination with the substitution in ParC (Ser80Ille) and occurred in ciprofloxacin-resistant isolates. In addition, some unusual genotypes were detected; strains containing only second-step mutations within GyrA were observed for Asp87Tyr (n = 4) and Asp87Asn (n = 1) and were associated with resistance to nalidixic acid. The substitution ParC(Ala85Ser) was observed in nalidixic acid–resistant *S. boydii* isolates with Gly(Ser80Leu) (n = 2) (Table 2). 

Our data document an ongoing trend toward dominance of *S. sonnei*, which is reflective of a current global shift in the epidemiologic distribution of this species (*10*). Of the 18 patients for whom travel to India was documented, isolates from 55.6% were resistant to ciprofloxacin, a finding that supports previous reports of importation of ciprofloxacin-resistant *Shigella* from India to Europe and the United States (*11*,*12*) and emphasizes the need to obtain travel information from patients receiving treatment for shigellosis. Furthermore, therapeutic efficiency of fluoroquinolones may be decreased because of the presence of PMQR determinants in phenotypically susceptible strains. PMQR genes are of concern because they not only promote mutations within the QRDR, resulting in resistance to fluoroquinolones, but they may disseminate among other species of *Enterobacteriaceae*.

Besides ciprofloxacin, the third-generation cephalosporin ceftriaxone is recommended as an alternative for the treatment of shigellosis (*1*). Resistance to the broad-spectrum β-lactam ampicillin was observed in all *Shigella* species (Table 1); however, the ESBL phenotype (resistance to cefotaxime; Table 1) was restricted to *S. sonnei* and was found in 8 strains (4.5% of *S. sonnei* isolates). PCR analysis confirmed the presence of *bla*_CTX-M_ genes in all 8 isolates: *bla*_CTX-M-3_ (n = 1), *bla*_CTX-M-14_ (n = 2), and *bla*_CTX-M-15_ (n = 5) (Table 2). The establishment of *bla*_CTX-M_–harboring *Shigella* as an additional reservoir of these widely disseminated resistance determinants poses a threat to the treatment of shigellosis, especially because all ESBLs detected in this study were CTX-M enzymes, which are also potent ceftriaxone hydrolyzers (*13*).

Screening of the 344 *Shigella* isolates for the presence of *mph(A)* revealed 7 (2%) positive strains: 2 *S. sonnei* and 5 *S. flexneri* (Table 2). *Shigella* species exhibiting reduced susceptibility to azithromycin are of great concern because azithromycin, in combination with colistin, has recently been found to represent a potentially invaluable option for the treatment of gram-negative rods expressing MDR, including carbapenem-resistant isolates of *Pseudomonas aeruginosa*, *Klebsiella pneumoniae*, and *Acinetobacter baumannii *(*14*). Hence, judicious use of this particular drug and susceptibility monitoring are warranted. Furthermore, our data show that *mph(A)* may be present in isolates displaying MICs as low as 12 μg/mL, highlighting the urgency with which azithromycin susceptibility breakpoints and interpretive criteria for *Enterobacteriaceae* are needed.

## Conclusions

Treatment of shigellosis with currently recommended antimicrobial drugs is increasingly threatened by the emergence of ciprofloxacin resistance, ESBLs, or plasmid-mediated azithromycin resistance in multidrug-resistant *Shigella* isolates. Because azithromycin is a last-resort antimicrobial agent used to treat shigellosis, the emergence of *mph(A)* among *Shigella* spp. is cause for concern.
